# Stereoselectivity Control Interplay in Racemic Lactide Polymerization by Achiral Al‐Salen Complexes

**DOI:** 10.1002/marc.202400733

**Published:** 2024-10-22

**Authors:** Serena Moccia, Massimo Christian D’ Alterio, Eugenio Romano, Claudio De Rosa, Giovanni Talarico

**Affiliations:** ^1^ Department of Chemical Sciences Università degli Studi di Napoli Federico II via Cintia Napoli 80126 Italy; ^2^ Scuola Superiore Meridionale Largo San Marcellino 10 Napoli 80138 Italy; ^3^ Scuola Normale Superiore Piazza dei Cavalieri 7 Pisa 56126 Italy

**Keywords:** chain end stereocontrol, DFT calculations of lactide polymerization, ligand wrapping mode, ROP of rac‐LA, stereoselective ring opening polymerization

## Abstract

The origin of stereocontrol in ring opening polymerization (ROP) of racemic lactide (*rac*‐LA) promoted by achiral aluminium‐based catalysts has been explained through DFT calculations combined with a molecular descriptor (%*V*_Bur_) and the activation strain model (ASM‐NEDA) analysis. The proposed chain end control (CEC) model suggests that the ligand framework adopts a chiral configuration mimicking the enantiomorphic site control (ESC) while also incorporating control of the last inserted monomer unit. It is found that the ligand wrapping mode around the aluminium centre is dictated by the monomer configuration (*R*,*R*‐LA and *S*,*S*‐LA). A good correlation with experimental data is achieved only when accounting for the ligand dynamic features and its steric influences, as highlighted by %*V*_Bur_ steric maps and ASM‐NEDA analysis. Understanding the ESC and CEC interplay is an important target for obtaining stereoselective ROP polymerization for the synthesis of biodegradable materials with tailored properties.

## Graphical abstract

The stereoselectivity control in the racemic lactide polymerization process, mediated by achiral Al‐Salen complexes, has been rationalized using DFT calculations combined with activation strain model analysis and a steric molecular descriptor (%*V*_Bur_). The primary features of both enantiomorphic site control and chain‐end control mechanisms are elucidated to explain the formation of isotactic [PDLA‐PLLA] stereoblocks.

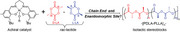

## 1 Introduction

Poly(lactic acid) (PLA) has gained significant relevance as a thermoplastic material due to its biocompatibility and biodegradability.^[^ ^1^ ^]^ Its intriguing mechanical and physical properties make PLA a good candidate to replace traditional petroleum‐based polyolefins,^[^ ^2^ ^]^ with the principles of the circular economy.^[^ ^3^ ^]^ Many efforts have been directed to better understand the correlation between PLA microstructures and material properties with targeted physical/mechanical properties, comparable to those of traditional plastics.^[^ ^4^ ^]^

**Chart 1:**
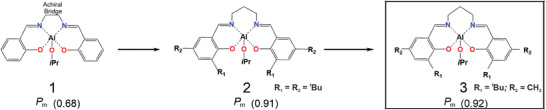
Achiral Al‐based systems reported by Spassky^[^ ^19^ ^]^ (**1**), Nomura^[^ ^20^ ^]^ (**2**) and Tong^[^ ^21^ ^]^ (**3**) for stereoselective *rac*‐LA ROP by CEC.

The two stereogenic centers on the cyclic monomer result in three different diastereoisomers, D‐LA (*R*,*R*), L‐LA (*S*,*S*), and *meso*‐LA (*R*,*S*). The mixture of homochiral polymers, PLLA and PDLA, co‐crystallize in a stereocomplex phase that exhibits enhanced thermal and mechanical properties in comparison to those of the homochiral phase.^[^ ^1^ ^]^ This leads to an increasing research interest in the stereoselective ROP of racemic lactide (*rac*‐LA),^[^ ^5^ ^]^ an equimolecular mixture of *R*,*R*‐LA and *S*,*S*‐LA, for the formation of isotactic‐diblock, and isotactic‐multiblock PLA,^[^ ^6^, ^7^ ^]^ materials with better performance. The most common route to achieve stereoselective PLA is through a metal catalyst,^[^ ^8^, ^9^, ^10^, ^11^, ^12^, ^13^, ^14^, ^15^ ^]^ although interesting examples of enantiomeric synthesis of *rac*‐LA by organocatalysis have been reported.^[^ ^16^, ^17^, ^18^ ^]^ From a mechanistic point of view, stereoselectivity can be governed by two different mechanisms: the enantiomorphic site control (ESC) and the chain‐end control (CEC). In the former stereoselectivity is dictated by the chirality of the catalyst whereas in the latter by the configuration of the last inserted monomeric unit. In CEC the catalyst framework is assumed to be generally achiral, although, in principle, the ligand framework may influence the stereogenic center of the last inserted monomer, which in turn determines the relative sequence of stereocenters in the main chain. The CEC stereocontrol is conceptually simple, and it can be argued that only a modest degree of chain‐end control can be achieved (measured by *P*_m_ parameter = probability to give *meso* enchainment). Indeed, the first achiral Al‐salen type complexes (system **1**, **Chart** **1**) introduced by Spassky shows a low stereocontrol (*P*_m_ = 0.68) with a formation of isotactic stereoblocks [PDLA‐PLLA]_n_ with a melting temperature (*T_m_*) = 149–151 °C.^[^ ^19^ ^]^ A notable exception was later reported by Nomura^[^ ^20^ ^]^ by using achiral aluminium‐Schiff base derivative (system **2**, Chart 1) with a relevant stereocontrol (*P_m_* = 0.91) and producing isotactic stereoblocks with improved thermal properties (*T_m_* = 192 °C). Recent developments in machine learning methods explored the multidimensional structure‐activity relationships for stereoselective achiral Al complexes through a Bayesian optimization procedure.^[^ ^21^ ^]^ In particular, the Al salen‐type achiral complex (system **3**, Chart 1) produces PLA stereoblocks with high *P_m_* = 0.92 and *T_m_* = 192 °C through CEC, as confirmed by ^1^H NMR analysis.^[^ ^21^ ^]^ Despite the interest in stereoselective ROP of *rac*‐LA, computational models rationalizing the origin of the stereoselectivity in such reactions are limited^[^ ^22^, ^23^, ^24^, ^25^, ^26^ ^]^ and focused primarily on the ESC. As a matter of fact, we recently reported that tracing the origin of stereocontrol in the *rac*‐LA ROP by enantiopure Al‐chiral catalysts is complicated by the variation of ligand wrapping modes *during* the ROP catalytic cycle.^[^ ^27^, ^28^, ^29^ ^]^ Nonetheless, computational studies on the chemical features of CEC governing stereoselectivity in *rac*‐LA polymerization are rare.^[^ ^30^, ^31^ ^]^ Intrigued by the (remarkable) level of stereocontrol achieved by system **3**,^[^ ^21^ ^]^ we used the Density Functional Theory (DFT) approach (computational details in Supporting Information) employed in chiral Al‐Salen catalysts^[^ ^27^, ^28^, ^32^ ^]^ to extract unprecedented chemical features for CEC of *rac*‐LA ROP promoted by **3**.

In this work, we will show that CEC and ESC are strictly interconnected and share similar chemical features. Indeed, although the catalyst precursor is achiral, the active site during the polymerization adopts a chiral wrapping mode due to the octahedral geometry originated from the coordination of the LA monomer. The DFT calculations have been combined with a molecular descriptor (%*V*_Bur_)^[^ ^33^, ^34^, ^35^ ^]^ and with the activation strain model (ASM),^[^ ^36^, ^37^ ^]^ coupled with natural energy decomposition analysis (NEDA)^[^ ^38^ ^]^ (see Supporting Information). This combined approach has been used in stereoselective olefin polymerization,^[^ ^39^, ^40^, ^41^, ^42^ ^]^ but there are no examples in literature for stereoselective *rac*‐LA ROP catalysis to the best of our knowledge.

## 2 Results and Discussion

**Figure 1:**
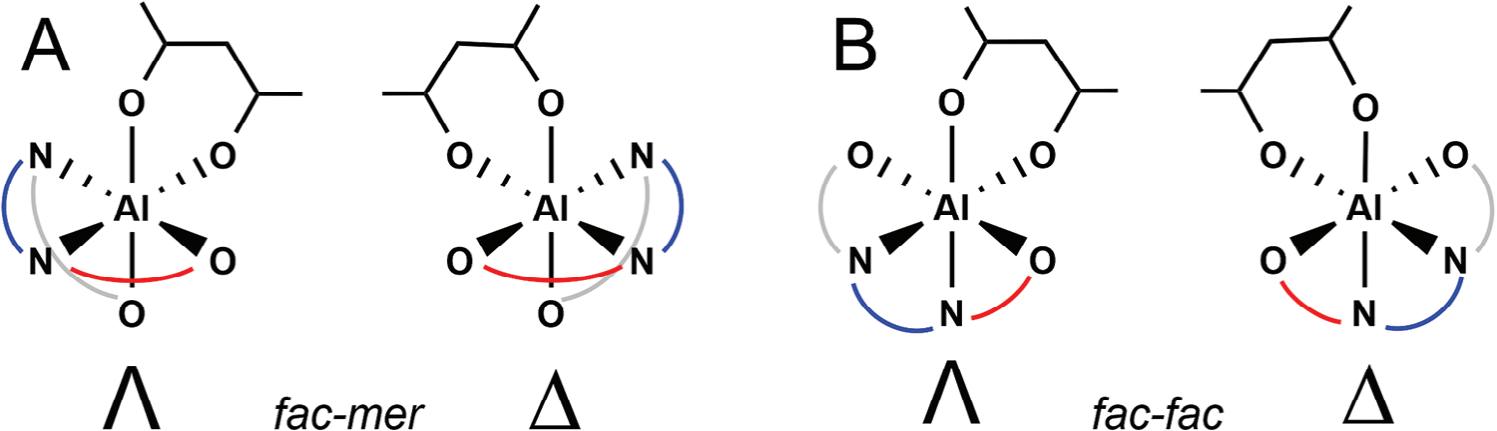
Λ and Δ enantiomers for the acac^−^ bidentate coordination at Al‐complex with *fac‐mer* and *fac‐fac* geometry.

The combination of all the elements of chirality considered here for stereoselective ROP of *rac*‐LA promoted by **3,** including the ligand wrapping mode, the *R*,*R* and *S*,*S*‐LA diastereoisomers and their enantiofaces, the chirality of growing chains and of the metal Al center are summarized in Figure S1 (Supporting Information). We preliminary calculated the wrapping mode energetic preference for **3** characterized by a *fac‐fac* (*ff*) and *fac‐mer* (*fm*) isomerism by using the bidentate acetylacetonate (acac^−^) coordination (**Figure** **1**).

**Figure 2:**
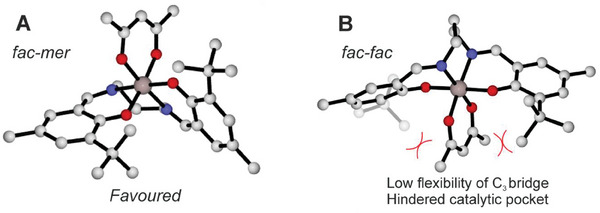
DFT optimized geometries for acac^−^ coordination at system **3** with *fm* (A) and *ff* (B) wrapping modes. The Gibbs energy preference of **A** versus **B** is 5.5 kcal mol^−1^.

Indeed, both wrapping modes resulted to be isoenergetic for *rac*‐LA polymerization with chiral salen Al‐catalyst;^[^ ^27^ ^]^ however, for the achiral system **3** we found a clear preference (5.5. kcal/mol) for *fm* configuration (**Figure** **2**A) with respect to *ff* (Figure 2B). The C_3_‐carbon bridge leads to a conformational strain keeping the O‐Al‐O angle at 180°, reducing significantly the size of the catalytic pocket for *ff* wrapping mode that will not be further considered in our ROP mechanistic study.

**Scheme 1:**
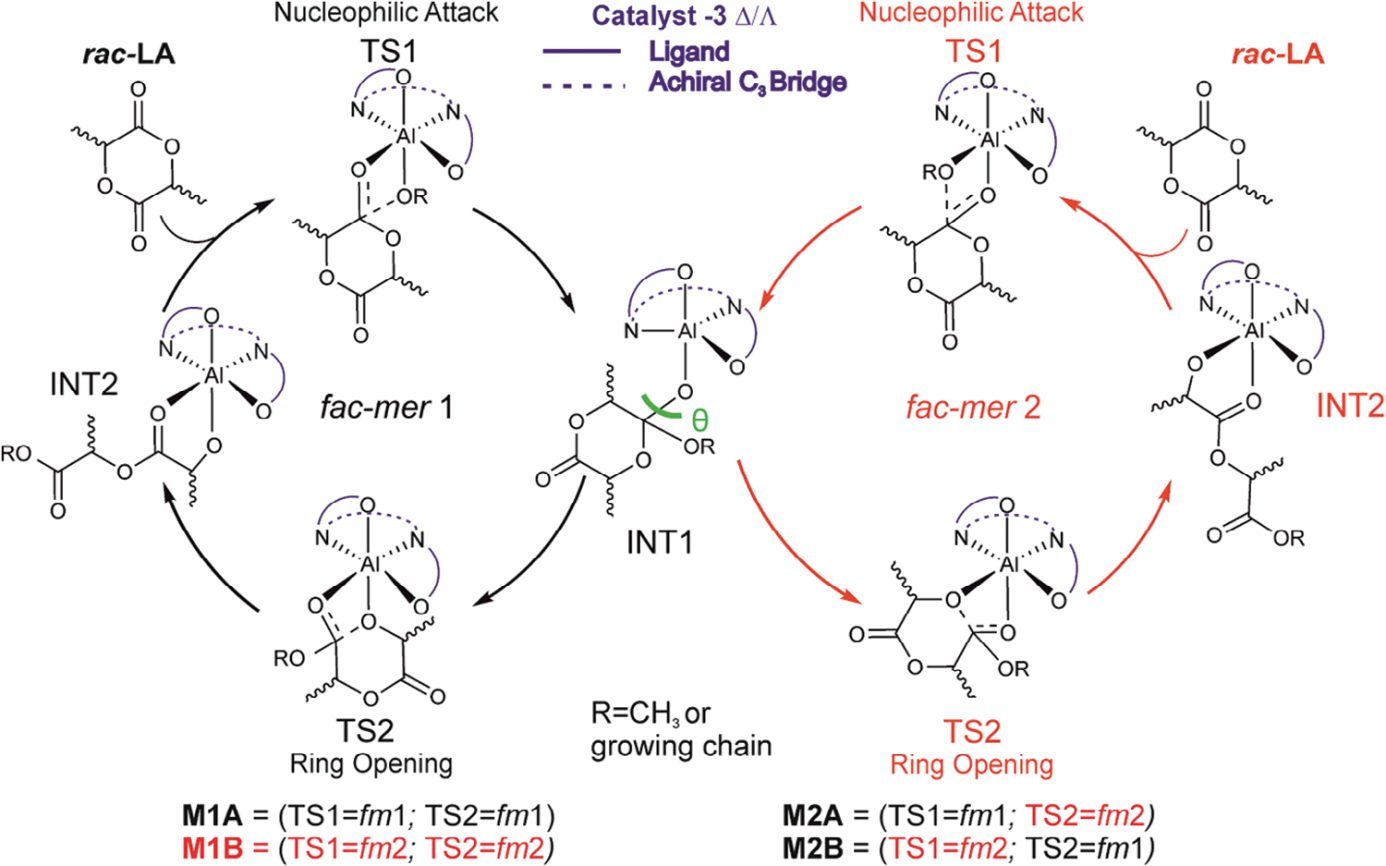
Coordination‐insertion mechanisms for *rac‐*LA ROP displayed by achiral‐Al systems with *fm*1 and *fm*2 wrapping modes.

Replacing the symmetric acac^−^ bidentate ligand with LA monomer and polymer growing chain splits *fac‐mer* isomer in two configurations, *fac‐mer*1 (*fm*1) and *fac‐mer*2 (*fm*2) (**Scheme** **1**) which differ for the position of methoxide/chain and monomer. This asymmetry needs to be carefully addressed in ROP processes, and we propose a coordination‐insertion mechanism substantially based on two catalytic cycles (Scheme 1). We defined the first nucleophilic attack of the carbonylic group of the monomer to the methoxy oxygen as the first transition state (TS1) followed by a first intermediate (INT1) and a second transition state (TS2) involving the ring opening of the cyclic monomer. After the formation of a second intermediate (INT2), the catalytic cycle repeats. However, due to the asymmetry of the two coordination sites generated by the monomer and the growing chain (*fm*1 and *fm*2), two possible mechanisms have been taken into account: **M1** (**A** and **B**) in which both TS1 and TS2 occur in the same wrapping mode (*fm*1 or *fm*2 respectively) and mechanism **M2** (**A** and **B**) in which TS1 and TS2 occur with different wrapping mode (TS1 is *fm*1 or *fm*2 and TS2 is *fm*2 or *fm*1, respectively).

**Figure 3:**
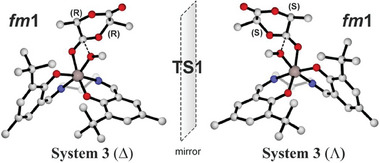
Δ and Λ enantiomers for LA nucleophilic addition (TS1) with a *fm*1 configuration for system **3**.

We selected the system **3** in Δ configuration recalling that results of Λ isomer are obtained by inversion of chirality as shown in **Figure** **3**.

**Figure 4:**
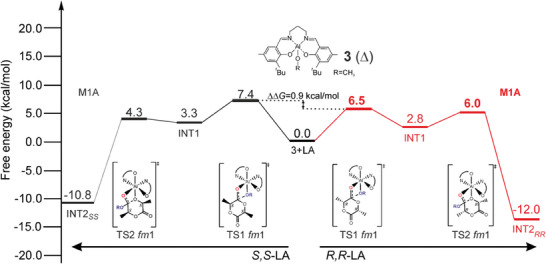
Gibbs energies for MEPs of *R,R*‐LA and *S,S*‐LA insertions into the Al‐OCH_3_ bond for system **3** (Δ)**. **The low lying path is marked in bold red (**M1A**).

In **Figure** **4** we report the Gibbs energies for the simplified minimum energy paths (MEP) for LA insertion (*R,R*‐LA on right and *S,S*‐LA on left side) into the Al‐OCH_3_ bond. Both *fm*1 and *fm*2 wrapping modes have been investigated, (complete lists in Tables S1 and S2, Supporting Information) by fixing a Δ octahedral chirality. These data cannot be compared with the experimental results due to the lack of chiral chain, nevertheless they can give hints on the interplay of the ligand wrapping modes. We found a small stereoselectivity (0.9 kcal mol^−1^) for *R*,*R*‐LA insertion, and the preferred paths show a *fm*1 wrapping modes for both the monomers with the **M1A** mechanisms (Figure 4).

**Figure 5:**
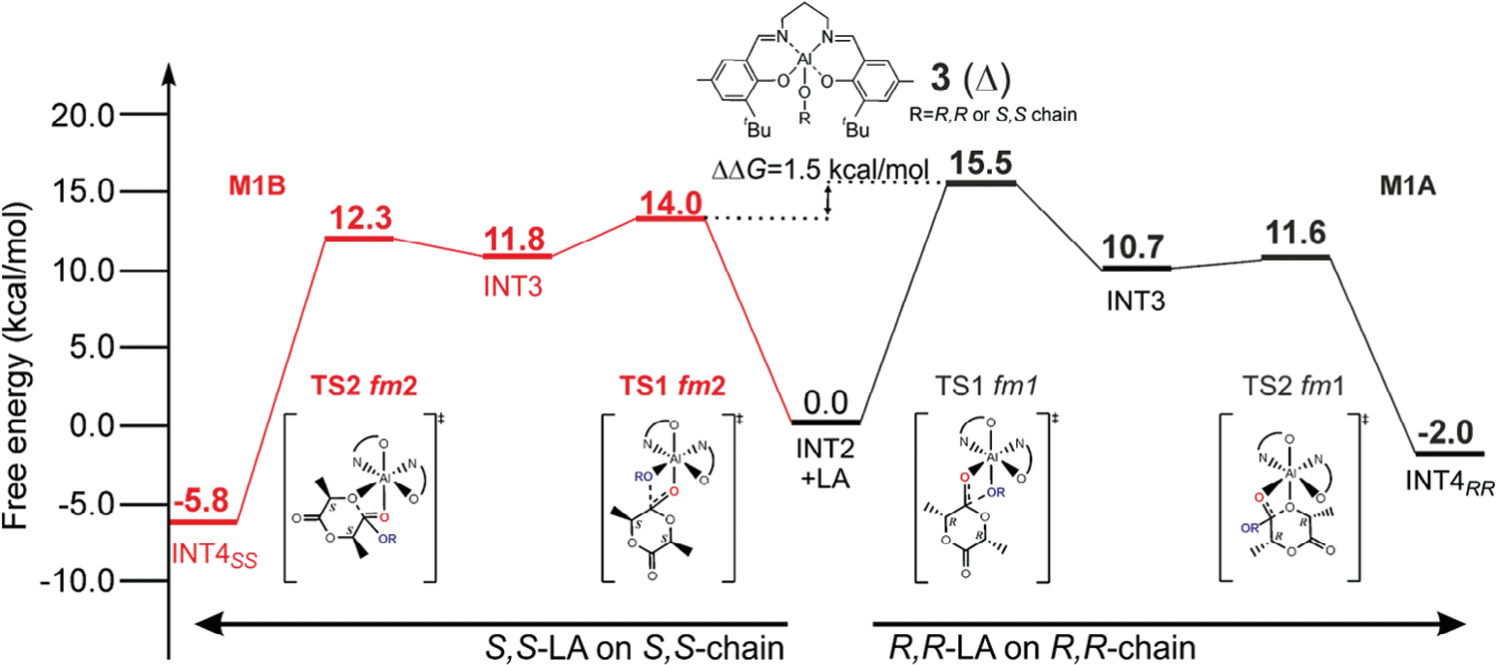
Gibbs energies for MEPs of *R,R*‐LA and *S,S*‐LA propagations for system **3** (**Δ**)**. **The low lying path is marked in bold red (**M1B**).

**Figure 6:**
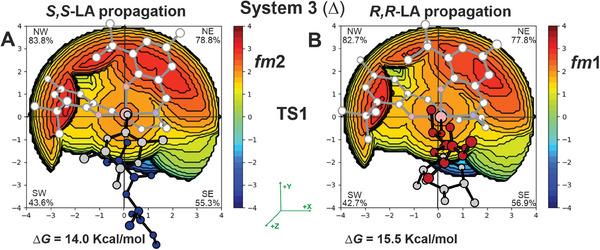
%*V*_Bur_ steric maps of the RLS for *S*,*S*‐LA propagation with a A) *fm*2 wrapping mode and *R*,*R*‐LA propagations with a B) *fm*1 wrapping mode promoted by system **3** with Δ chirality.

The computed Gibbs energies of the simplified MEPs (complete lists in Tables S3 and S4, Supporting Information) by using chiral growing chains for *R*,*R*‐LA (**Figure** **5**‐right) and *S*,*S*‐LA (Figure 5‐left) propagations provide greater insight. Notably, we predicted distinct mechanisms for *S,S‐*LA propagation, which follows the **M1B** pathway (*fm*2 catalyst geometry in both TS1 and TS2). In contrast, *R,R‐*LA propagation favors the **M1A** mechanism (*fm*1 catalyst geometry in both TS1 and TS2). The TS1s are the rate‐limiting steps (RLS) and the calculated stereoselectivity of 1.5 kcal mol^−1^ (ΔΔ*G*, Figure 5) is in good agreement with the experimental results.^[^ ^21^ ^]^ However, the underlying reason for the preference for *S*,*S*‐LA propagation requires further investigation. The buried volume analysis (%*V*_Bur_) on the RLS shows that *S,S‐*chain at *fm*2 (**Figure** **6**A) is positioned in the less hindered zone of the SE quadrant along the negative y‐*axis*, while the *R,R‐*chain at *fm*1 (Figure 6B) is pointing toward the positive z‐*axis*, that is the overcrowded area of SE quadrant where are located the *^t^* Bu substituents.

The optimized DFT geometries for the two RLS are reported in Figure S1 (Supporting Information) where, in addition to the ligand steric effects, we note also that the monomer conformations show the methyl groups in equatorial position for *S,S‐*LA (Figure S1A, Supporting Information) and in axial position for *R,R‐*LA (Figure S1B, Supporting Information).

For achieving a more complete picture on the origin of the stereoselectivity and on the variation of the energetics depending on ligand wrapping modes, we used the ASM‐NEDA analysis^[^ ^36^, ^37^, ^38^ ^]^ (see Supporting Information for details) and the results are reported in **Table** **1**.

**Table 1:** ASM‐NEDA analysis for isotactic enchainments for ROP polymerization of *rac*‐LA promoted by system **3**. Values are reported in kcal/mol and with respect to the low‐lying transition states.

Entry	Assembly mode	Wrapping mode	ΔΔ*G*	ΔΔ*E*_Tot_	ΔΔ*E*_Strain_	ΔΔ*E*_Int_	ΔΔ*E*_Strain(Cat)_	ΔΔ*E*_Strain(Mon)_
**1**	*S,S‐*LA on *S,S‐*chain	*fm*2	**1.5**	1.3	11.8	−10.5	6.8	5.0
	*R,R‐*LA on *R,R‐*chain	*fm*1						
**2**	*R,R‐*LA on *R,R‐*chain	*fm*1	**2.6**	4.6	11.1	−6.5	4.5	6.6
	*R,R‐*LA on *R,R‐*chain	*fm*2						
**3**	*S,S‐*LA on *S,S‐*chain	*fm*2	**3.1**	1.3	23.6	−22.3	10.7	12.9
	*S,S‐*LA on *S,S‐*chain	*fm*1						

In Table 1 the energetic variation of the different assembly modes obtained by the ASM‐NEDA analysis (ΔΔ*E*_Tot_, Table 1) has been decomposed into the two components ΔΔ*E*_Strain_ and ΔΔ*E*_Int_. Following our previous experiences on the olefin polymerization transition metal catalyzed^[^ ^39^, ^40^, ^41^, ^42^ ^]^ we further decomposed the ΔΔ*E*_Strain_, into two fragments corresponding to the ligand catalyst + growing chain (Δ*E*_Strain(Cat)_) and to the monomer (Δ*E*_Strain(Mon)_). The ASM‐NEDA values do not include solvent and entropic corrections, so discrepancies with the calculated DFT Gibbs energies (ΔΔ*G*, Table 1) are expected. The energetic difference between the RLS TSs of the two isotactic propagations, namely *S*,*S*‐LA on *S*,*S* chain and *R*,*R*‐LA on *R*,*R* chain (entry 1 in Table 1), is positive indicating a preference for the former. The Δ*E*_Strain(Cat)_ and Δ*E*_Strain(Mon)_ reveal that the catalyst with *S,S‐*ending chain in *fm*2 geometry is less strained compared to its *R*,*R*‐ending chain counterpart in the *fm*1 geometry.

This supports our interpretation of the %*V*_Bur_ maps of Figure 6 where the *R,R*‐chain is positioned in the SE red zone, leading to a more congested environment for both the chain/ligand framework.

Additionally, the Δ*E*_Strain(Mon)_ values reflect the different monomer conformations, with the methyl groups in equatorial and axial orientations for *S*,*S*‐LA and *R*,*R*‐LA, respectively, stabilizing the *S*,*S*‐LA conformation (Figure S1, Supporting Information). The calculated energetic preference for the *fm*1 conformation in *R*,*R*‐LA propagation (entry 2, Table 1) and for the *fm*2 conformation in *S*,*S*‐LA propagation (entry 3, Table 1) further highlights the influence of steric effects on both the catalyst fragment and monomer deformation (Figure S2, Supporting Information). A complete analysis of the ΔΔ*E*_Int_ contributions is provided in Table S5 (Supporting Information).

**Figure 7:**
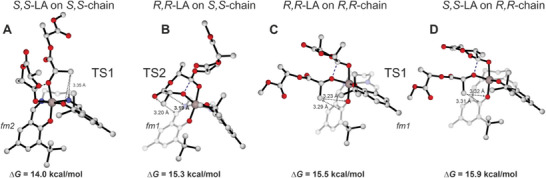
TSs geometries for RLSs at system **3** of *S*,*S*‐LA insertions within the A) *S*,*S* and B) *R*,*R*‐ending chains, and C) *R*,*R*‐LA insertions within the *R*,*R* and D) *S*,*S*‐ending chains. The RLSs correspond to A,C,D) TS1 and B) TS2. H atoms omitted for clarity. Distances in Å.

Finally, we decided to further investigate the role played by the chiral growing chain by considering different assembly modes, thus including the *S*,*S* and *R*,*R*‐LA insertions on system **3** with *R*,*R* and *S*,*S*‐ending chains, respectively. The key energetic results are reported in **Table** **2** (complete lists in Tables S6 and S7, Supporting Information) and the DFT optimized geometries of the RLS TSs are reported in **Figure** **7**. Interestingly, we found that *S,S*‐LA insertion is favored over *R,R*‐LA into the *S,S*‐chain; however, this preference is reversed when considering the *R*,*R*‐LA last inserted unit (thus forming the *R*,*R*‐ending chain), in line with the CEC (Table 2).

**Table 2:** Gibbs activation energies (Δ*G* ^‡^_RLS_) for the RLS and Gibbs activation energy differences (ΔΔ*G*) relative to the MEP for *R,R*‐LA and *S,S*‐LA insertions depending on the assembly mode (values in kcal mol^−1^).

Assembly mode	Δ*G* ^‡^_RLS_ [kcal mol^−1^]	ΔΔ*G* [kcal mol^−1^]
*S,S‐*LA on *S,S* chain	14.0 (TS1)	0.0
*R,R‐*LA on *S,S* chain	15.3 (TS2)	1.3
*R,R‐*LA on *R,R* chain	15.5 (TS1)	1.5
*S,S‐*LA on *R,R* chain	15.9 (TS1)	1.9

**Scheme 2:**
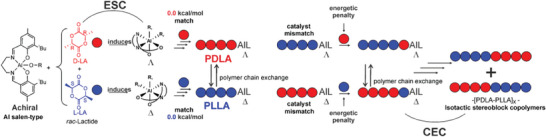
Proposed mechanism for the formation of PLA isotactic stereoblock copolymers by CEC.

At this stage, it is worth to resume the kinetic experiments reported by Nomura^[^ ^18^ ^]^ on similar achiral Al‐salen catalysts where the polymerization kinetic constants for the enantiopure LLA are higher than the ones for *rac*‐LA (Figure S3, Supporting Information). The values reported in Table 2 match the experimental data (Supporting Information), suggesting that Δ configuration exhibits a clear preference toward the *S,S‐*LA (Λ configuration toward the *R,R‐*LA); the following insertion of *R*,*R*‐LA forming an *R*,*R*‐ending chain makes a mismatch^[^ ^43^ ^]^ for the catalyst with an increasing of the activation Gibbs energies (Table 2). This mechanism, which mixes the features of the ESC (chiral configuration with the *fm* wrapping mode) and the CEC (inverted preference for *R*,*R* and *S*,*S*‐LA depending on the last inserted unit), is a reasonable way to explain the isotactic stereoblock microstructure and it is summarized in **Scheme** **2**. Incidentally, the Scheme 2 may also account for the formation of highly isotactic stereoblock copolymer of PLA via the polymer chain exchange mechanism proposed by Coates^[^ ^44^ ^]^ where the two preferred Δ/Λ configurations (Figure 3) may exchange the *S,S and R,R*‐ ending chains after a certain number of insertions.

For the final validation of our computational analysis, we simulated the ROP of *rac*‐LA promoted by the achiral system **1** (Chart 1), which shows a lower experimental stereoselectivity (*P*_m_ = 0.68) compared to system **3**. Our results confirm that the key features described previously – *fm*2 preference for *S*,*S*‐LA and *fm*1 preference for *R*,*R*‐LA‐ are maintained, with the calculated stereoselectivity (ΔΔ*G* = 0.8 kcal/mol) in good agreement with the experimental data. Optimized geometries of system **1** for the *rac*‐LA TSs along with the %*V*_Bur_ steric maps are reported in Figure S4 (Supporting Information). The absence of *^t^* Bu substituents on the ligand reduces the steric hindrance in the SE quadrant leading to the observed decrease in stereoselectivity.

## 3 Conclusion

In this contribution, we explained, through DFT analysis combined with %*V*_Bur_ steric maps and the ASM‐NEDA analysis, the origin of stereocontrol in the ROP of *rac*‐LA promoted by achiral Al‐based catalysts. The mechanism(s) we proposed, as well as the methodology we used for explaining the stereoselective ROP by enantiopure complexes following the enantiomorphic site control,^[^ ^27^, ^28^, ^45^ ^]^ is working well also for stereoselective ROP by achiral Al‐based complex following the chain end control. We carefully analyzed all the elements of chirality during the polymerization processes and we identified the rate‐limiting steps for *R*,*R*‐LA and *S*,*S*‐LA propagation. We found that the ligand wrapping mode is dictated by the monomer configuration (*fm*2 for *S*,*S*‐LA and *fm*1 for *R*,*R*‐LA). It is worth to stress that the match with the experimental parameters (*P*_m_ and *T*_m_) is sorted out only by including the ligand dynamic features reported in Scheme 1. The main factors were revealed by the %*V*_Bur_ steric maps and the ASM‐NEDA analysis.

Furthermore, the values resulting from computing all the assembly modes agree with the polymerization kinetics of homochiral PLLA leading to a mechanistic path proposed in Scheme 2. In conclusion, we explained how achiral Al‐Salen systems dictate the stereoselectivity through a chain end control exerted by the chiral configuration of the active site. Revealing the origin of the stereocontrol is an important target to achieve stereoselective ROP polymerization for the synthesis of biodegradable materials with tailored properties.^[^ ^46^, ^47^, ^48^, ^49^, ^50^ ^]^

## Conflict of Interest

The authors declare no conflict of interest.

## Supporting information



Supporting Information

## Data Availability

The data that support the findings of this study are available in the supplementary material of this article.
