# Supplementary material for: Stereoselectivity Control Interplay in Racemic Lactide Polymerization by Achiral Al‐Salen Complexes
Source: Macromol Rapid Commun. 2024 Oct 22;46(3):2400733. doi: 10.1002/marc.202400733 (PMC11800063; doi:10.1002/marc.202400733)
Supplement: Supplementary file 1 — Supporting Information [file MARC-46-2400733-s001.pdf]

Macromolecular Rapid Communications

Supporting Information

for *Macromol. Rapid Commun.*, DOI 10.1002/marc.202400733

Stereoselectivity Control Interplay in Racemic Lactide Polymerization by Achiral Al-Salen Complexes

*Serena Moccia, Massimo Christian D' Alterio, Eugenio Romano, Claudio De Rosa and Giovanni Talarico**

Supporting Information

Stereoselectivity Control Interplay in Racemic Lactide Polymerization by Achiral Al-Salen Complexes

Serena Moccia,[1] Massimo Christian D'Alterio,[1] Eugenio Romano,[2,3] Claudio De Rosa,[1] Giovanni Talarico[1,2]

[1] Department of Chemical Sciences, Università degli Studi di Napoli Federico II, via Cintia, 80126 Napoli, Italy

[2] Scuola Superiore Meridionale, Largo San Marcellino 10, I-80138 Napoli, Italy

[3] Scuola Normale Superiore, Piazza dei Cavalieri 7, 56126 Pisa, Italy

E-mail: talarico@unina.it

Table of Contents

Computational Details S2
Scheme S1 S3
Tables S1-S2 S4
Tables S3-S4 S5
Figures S1-S2 S6
Figure S3 S7
Tables S5-S7 S8
Figure S4 S9
References S10

Computational Details

All the density functional theory (DFT) calculations were performed using the Gaussian16 package.[1] Geometry optimizations were performed using the B3LYP functional of Becke[2] with the triple-ζ basis set 6-311G(d,p)[3] for the metal Al and the standard split-valence basis set with a polarization function of Ahlrichs and coworkers (SVP keyword in Gaussian)[4] for H, N, O and C atoms. Geometry optimizations were performed without symmetry constraints. Transition states (TSs) were approached through a linear transit procedure using the forming or disappearing C–O bond as the reaction coordinate. All the geometries discussed in this work were confirmed as minima or TSs by frequency calculations. Finally, single-point (SP) energy calculations in solution were performed with the triple-ζ basis set 6-311G(d,p)[3] for Al, H, N, O and C. Solvent effects were included with the conductor-like polarizable continuum model (CPCM)[5] using toluene as solvent. The influence of the dispersion was also considered with the model D3BJ (B3LYP-D3BJ).[6] The Gibbs energy values of each structure were calculated considering the contribute of thermal and electronic energies with the related correction obtained by the SP energy calculation. All calculations have been optimized from two different initial octahedral coordination geometries[7] (Scheme S1, A), identified with the names: *fac-mer* 1 (*fm*1) and *fac-mer* 2 (*fm*2). The *fac-fac* (*ff*) geometry of the ROP mechanism was ruled out since it is too high in energy. The nucleophilic attack by the methoxidic oxygen was studied considering the attack on both the monomeric *re* and *si* enantiofaces (Scheme S1, B) for the cyclic monomers. In addition, we considered the chirality of the chain (Figure S1, C) and two different configurations adopted by the catalyst, *i.e.* Δ and Λ (Scheme S1, D).

The ASM model proposed by Bickelhaupt[8], is a fragment based approach in which the electronic energy is decomposed into two main contributions:

$$\Delta E_{\text{Tot}} = \Delta E_{\text{Strain}} + \Delta E_{\text{Int}}$$

ΔE_{Strain} is the energy needed to deform the reactant in order to reach the geometries required to them for reacting and ΔE_{Int} is the interaction energy related to the mutual interaction between the fragments. The former term is the sum of the strain related to each reactant: $\Delta E_{\text{Strain}} = \Delta E_{\text{Strain,frag1}} + \Delta E_{\text{Strain,frag2}}$. In this work, the fragment 1 corresponds to the catalytic active specie involving the growing polymer chain ($\Delta E_{\text{Strain(Cat)}}$), whereas fragment 2 is LA monomer ($\Delta E_{\text{Strain(Mon)}}$).

The ΔE_{Int} term has been calculated and decomposed using the NEDA (Natural Energy Decomposition Analysis) scheme[9] using the NBO version 7 software linked to Gaussian16.[10] This permits to obtain ΔE_{Int} and all its components, namely electrostatic, polarization, charge transfer, exchange and deformation components: $\Delta E_{\text{Int}} = \text{ES} + \text{POL} + \text{CT} + \text{XC} + \text{DEF}$ where ES = classical electrostatic interaction; POL = polarization interaction; CT = charge transfer based on interaction between filled and empty orbitals; XC = exchange-correlation interaction; DEF = the energy cost to distort a fragment wavefunctions in the field of all other fragments of the complex. Dispersion contributions have been also estimated by the D3BJ model of Grimme. The NEDA scheme, in addition to, works in the complete basis set of the complex, hence the reported energies are corrected for the Basis Set Superposition Error (BSSE) as proposed by Boys and Bernardi.[11]

In Figure S3 are reported the kinetic constant of L-LA and *rac*-LA polymerization published by Nomura[12] for an achiral Al system similar to the one analyzed in this study. The ratio between the kinetic constant gives a $\Delta\Delta G$ variation of 0.45 kcal/mol by using the Boltzmann distribution equation (Figure S3). If we approximate the kinetic constant for propagation (k_{p}) by using the equation reported by Nomura $k_{(rac)\text{p}} = (k_{(L\text{-}L)\text{p}} + k_{(D\text{-}L)\text{p}})/2$ and by comparing with the results reported in Table 3 of the main text, we estimated a calculated $\Delta\Delta G$ variation of 0.65 kcal/mol, in very good agreement with the experiments.

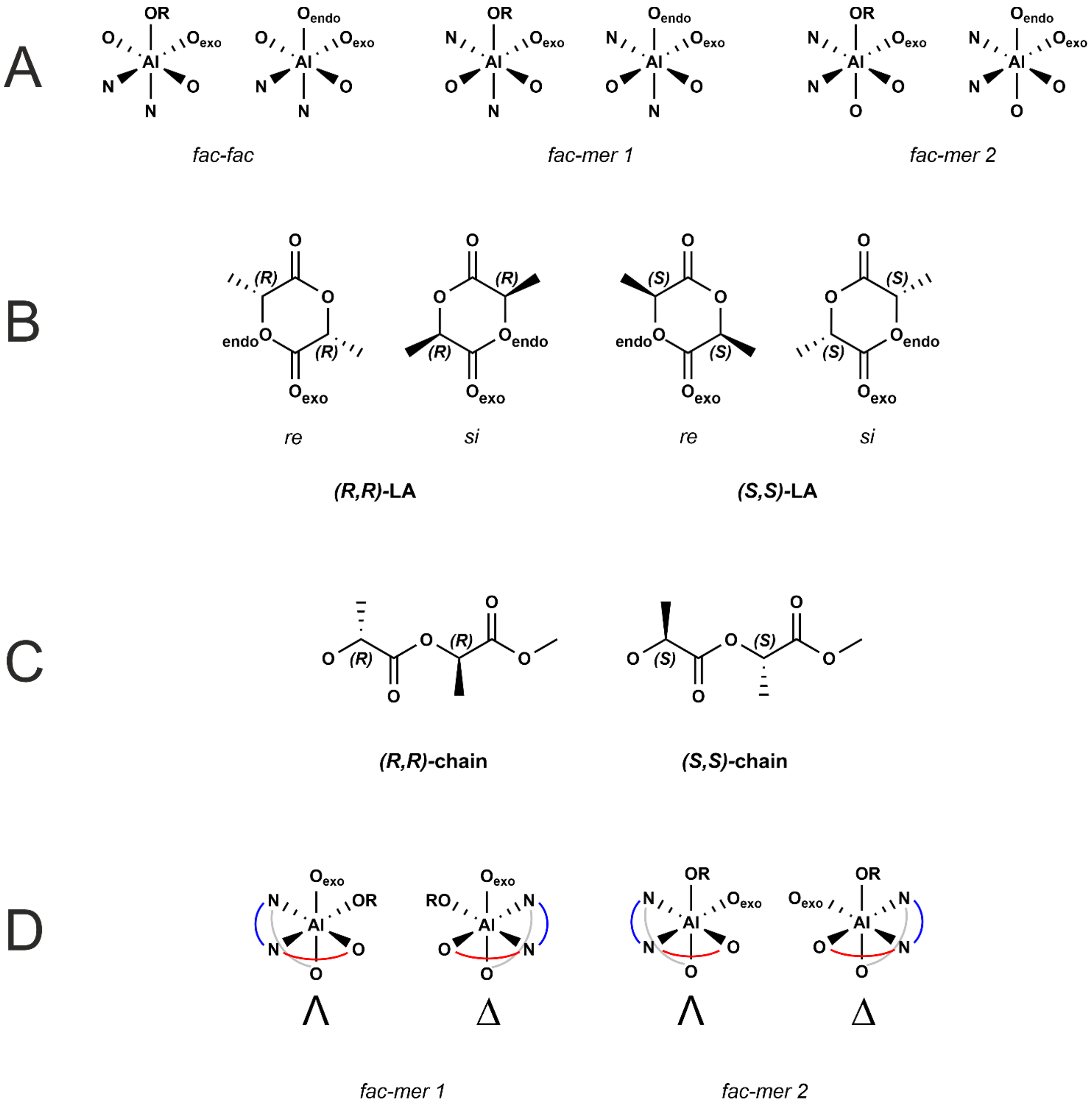

Scheme S1. List of elements of chirality considered in this work. With OR we called the OMe and with O_{endo} and/or O_{exo} we defined the endocyclic and exocyclic O of lactide (**A**); monomer chiralities (*RR*-LA and *SS*-LA) as well as the monomer enantiofaces (*re* and *si*) (**B**); chirality of the growing chain (*RR*-chain and *SS*-chain) (**C**); octahedral configuration for *fac-mer* 1 and *fac-mer* 2 wrapping mode during the ROP (**D**).

Table S1. TSs Gibbs energies (Δ*G*, in kcal/mol, with respect to system-**3** (Δ)) for all reaction paths computed for *R*,*R*-LA insertion into the Al-OCH_3 chain bond. Values calculated in toluene. In bold the low-lying paths.

Monomer Enantioface	*R,R* *re*			Monomer Enantioface	*R,R* *si*		
		**TS1**	**TS2**			**TS1**	**TS2**
Mechanism 1 (**M1**)	A	8.6	3.5	Mechanism 1 (**M1**)	A	**6.5**	**6.0**
	B	10.8	8.1		B	7.5	10.0
		**TS1**	**TS2**			**TS1**	**TS2**
Mechanism 2 (**M2**)	A	8.6	8.1	Mechanism 2 (**M2**)	A	6.5	10.0
	B	10.8	3.5		B	7.5	6.0

Table S2. TSs Gibbs energies (Δ*G*, in kcal/mol, with respect to system-**3** (Δ)) for all reaction paths computed for *S*,*S*-LA insertion into the Al-OCH_3 chain bond. Values calculated in toluene. In bold the low-lying paths.

Monomer Enantioface	*S,S* *re*			Monomer Enantioface	*S,S* *si*		
		**TS1**	**TS2**			**TS1**	**TS2**
Mechanism 1 (**M1**)	A	9.4	7.8	Mechanism 1 (**M1**)	A	**7.4**	**4.3**
	B	6.6	16.1		B	7.5	4.6
		**TS1**	**TS2**			**TS1**	**TS2**
Mechanism 2 (**M2**)	A	9.4	16.1	Mechanism 2 (**M2**)	A	7.4	4.6
	B	6.6	7.8		B	7.5	4.3

Table S3. TSs Gibbs energies (ΔG, in kcal/mol, with respect to (*R*,*R*)-**3-Δ** + INT3-*fac-mer*2) for all reaction paths computed for *R*,*R*-LA insertion into the Al-*R*,*R*-chain bond. Values calculated in toluene. In bold the low-lying paths.

Chain	*R,R*			Chain	*R,R*		
Monomer	*R,R*			Monomer	*R,R*		
Enantioface	*re*			Enantioface	*si*		
		**TS1**	**TS2**			**TS1**	**TS2**
Mechanism 1 (**M1**)	A	**15.5**	**11.6**	Mechanism 1 (**M1**)	A	17.0	12.6
	B	18.1	16.6		B	23.6	17.1
		**TS1**	**TS2**			**TS1**	**TS2**
Mechanism 2 (**M2**)	A	15.5	16.6	Mechanism 2 (**M2**)	A	17.0	17.1
	B	18.1	11.6		B	23.6	12.6

Table S4. TSs Gibbs energies (ΔG, in kcal/mol, with respect to (*S*,*S*)-**3-Δ** + INT3-*facmer2*) for all reaction paths computed for *S*,*S*-LA insertion into the Al-*S*,*S*-chain bond. Values calculated in toluene. In bold the low-lying paths.

Chain	*S,S*			Chain	*S,S*		
Monomer	*S,S*			Monomer	*S,S*		
Enantioface	*re*			Enantioface	*si*		
		**TS1**	**TS2**			**TS1**	**TS2**
Mechanism 1 (**M1**)	A	17.1	14.0	Mechanism 1 (**M1**)	A	19.8	23.6
	B	**14.0**	**12.3**		B	12.3	22.5
		**TS1**	**TS2**			**TS1**	**TS2**
Mechanism 2 (**M2**)	A	17.1	12.3	Mechanism 2 (**M2**)	A	19.8	22.5
	B	14.0	14.0		B	12.3	23.6

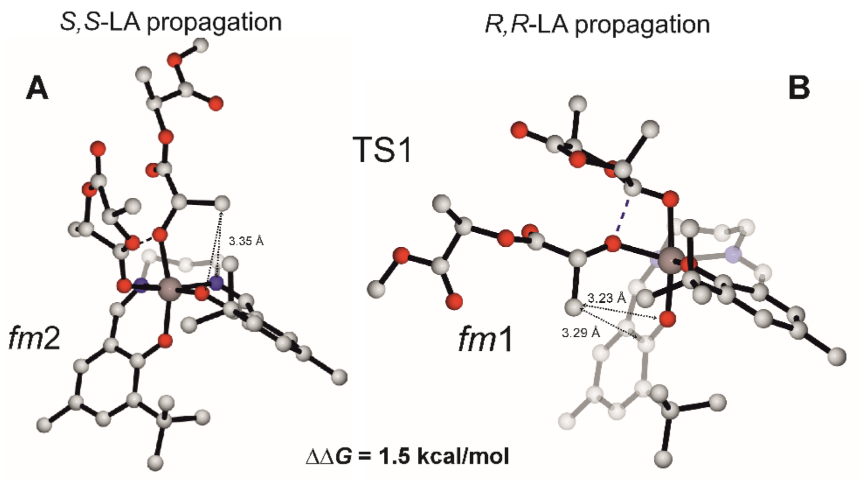

Figure S1. TSs geometries for RLS of *S*,*S*-LA (**A**) and *R*,*R*-LA (**B**) propagations promoted by system **3** corresponding to TS1 with a *fm*2 (**A**) and *fm*1 (**B**) wrapping modes. H atoms omitted for clarity. Distances in Å.

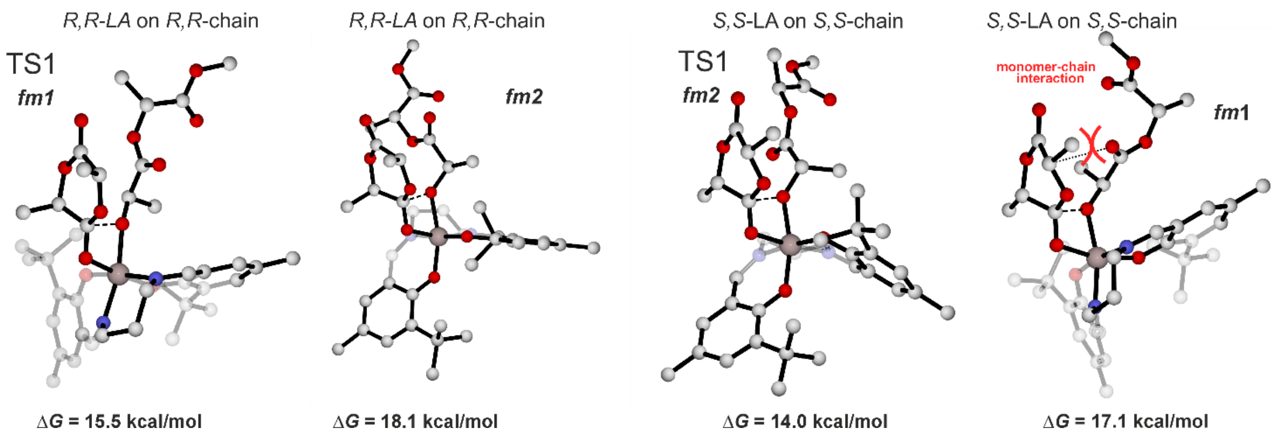

Figure S2. TSs geometries and Gibbs energies explaining the preference for *fm*1 and *fm*2 wrapping modes for *R*,*R*-LA and *S*,*S*-LA propagations, respectively. H atoms omitted for clarity.

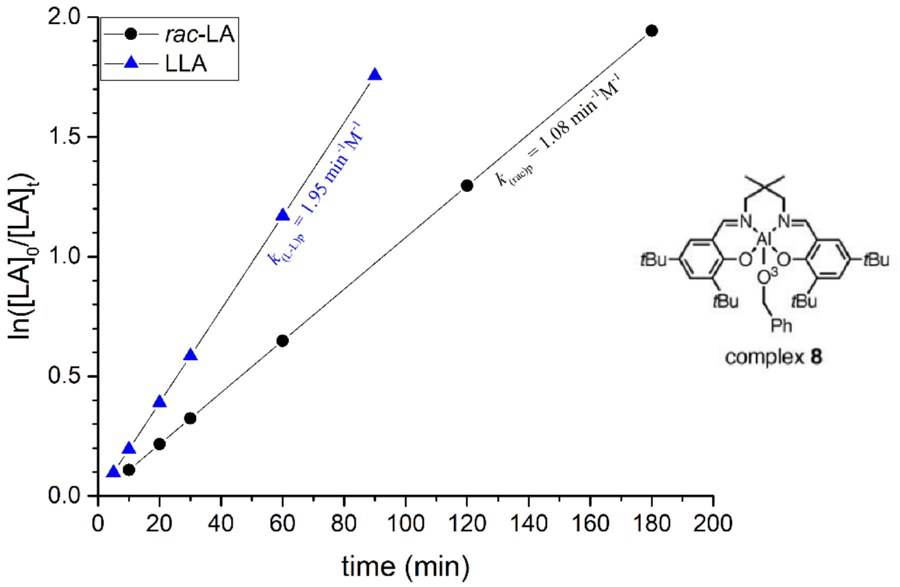

Figure S3. Kinetic studies of LLA and *rac*-LA using complex **8** reported by Nomura.[12] Polymerization conditions: toluene; 70°C; $[LLA]_0$=[*rac*-LA]$_0$=1.0 M; $[LA]_0/[Al]_0$=100.

Table S5. Complete NEDA analysis decomposition for ΔE_{Int} (ΔE_{Int} = EL + CORE + CT) for RLS TSs and the opposite isotactic enchainment. The ΔE_{Int} values are reported with dispersion contribute.

System	CT	ES	POL	XC-E(D3)	E(D3)	DEF (Cat)	DEF (Mon)	SE (Cat+Mon)	ΔE_{Int}
S,S-LA on *S,S*-chain (*fm*2)	-190.0	-93.4	-78.9	-43.6	-19.8	163.8	225.6	42.1	-36.2
R,R-LA on *R,R*-chain (*fm*1)	-217.5	-117.8	-96.6	-50.2	-18.0	195.9	257.4	52.3	-46.8
R,R-LA on *R,R*-chain (*fm*2)	-273.7	-137.2	-116.5	-54.0	-19.2	235.1	298.2	56.6	-53.3
S,S-LA on *S,S*-chain (*fm*1)	-284.0	-142.6	-102.5	-59.9	-19.7	257.2	307.0	64.1	-58.6

Table S6. TSs Gibbs energies (ΔG, in kcal/mol, with respect to (*R,R*)-**3** (Δ) + INT3-*fm*2) for all reaction paths computed for *S,S*-LA insertion into the Al-*R,R*-chain bond. Values calculated in toluene. In bold the low-lying paths.

Chain / Monomer / Enantioface	*R,R* / *S,S* / *re*			Chain / Monomer / Enantioface	*R,R* / *S,S* / *si*		
		**TS1**	**TS2**			**TS1**	**TS2**
Mechanism 1 (**M1**)	A	**15.9**	**11.0**	Mechanism 1 (**M1**)	A	15.4	23.5
	B	18.0	12.1		B	18.9	24.0
		**TS1**	**TS2**			**TS1**	**TS2**
Mechanism 2 (**M2**)	A	15.9	12.1	Mechanism 2 (**M2**)	A	15.4	24.0
	B	18.0	11.0		B	18.9	23.5

Table S7. TSs Gibbs energies (ΔG, in kcal/mol, with respect to (*S,S*)- **3** (Δ) + INT3-*fm*2) for all reaction paths computed for *R,R*-LA insertion into the Al-*S,S*-chain bond. Values calculated in toluene. In bold the low-lying paths.

Chain / Monomer / Enantioface	*S,S* / *R,R* / *re*			Chain / Monomer / Enantioface	*S,S* / *R,R* / *si*		
		**TS1**	**TS2**			**TS1**	**TS2**
Mechanism 1 (**M1**)	A	16.9	15.3	Mechanism 1 (**M1**)	A	15.8	11.6
	B	14.7	21.0		B	17.5	15.5
		**TS1**	**TS2**			**TS1**	**TS2**
Mechanism 2 (**M2**)	A	16.9	21.0	Mechanism 2 (**M2**)	A	15.8	15.5
	B	**14.7**	**15.3**		B	17.5	11.6

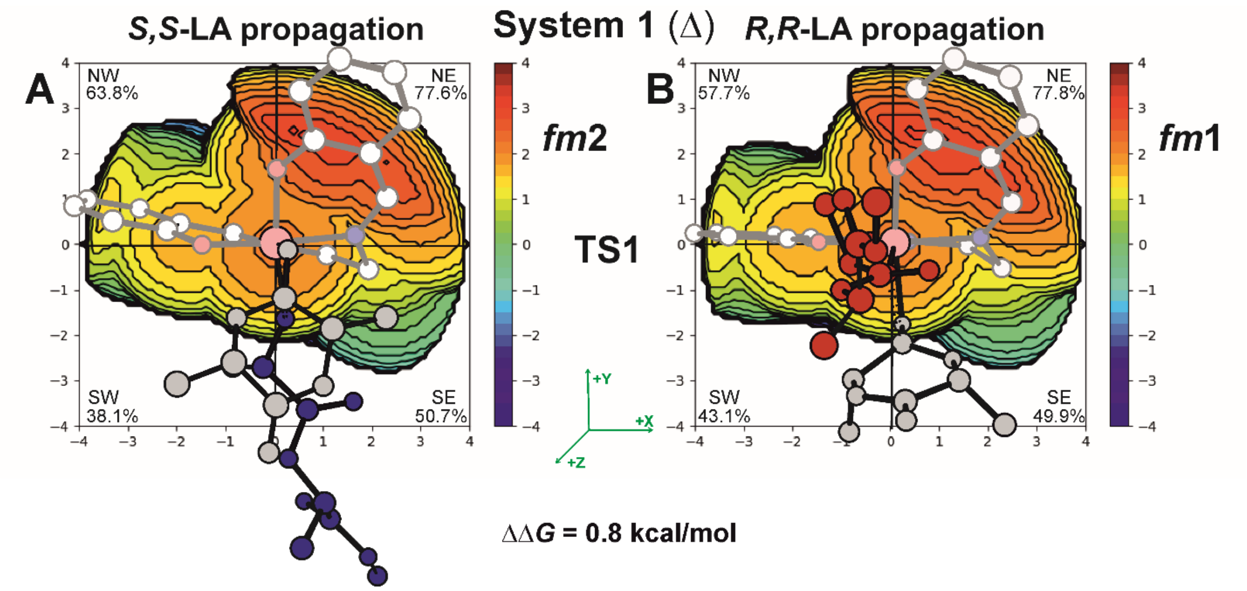

Figure S4. TSs geometries and %V_{Bur} steric maps for RLS of *S*,*S*-LA (**A**) and *R*,*R*-LA (**B**) propagations promoted by system **1** corresponding to TS1 with *fm*2 (**A**) and *fm*1 (**B**) wrapping modes. H atoms omitted for clarity.

References

[1] M. Frisch, G. Trucks, H. Schlegel, G. Scuseria, M. Robb, J. Cheeseman, G. Scalmani, V. Barone, G. Petersson, H. Nakatsuji, *Inc., Wallingford CT* **2016**, *2016*.

[2] A. D. Becke, *Phys. Rev. A* **1988**, *38*, 3098-3100.

[3] a) A. McLean, G. Chandler, *J. Chem. Phys.***1980**, *72*, 5639-5648; b) R. Krishnan, J. S. Binkley, R. Seeger, J. A. Pople, *J. Chem. Phys.* **1980**, *72*, 650-654.

[4] a) A. Schäfer, H. Horn, R. Ahlrichs, *J. Chem. Phys.***1992**, *97*, 2571-2577; b) T. H. Dunning Jr, P. J. Hay, in *Methods of electronic structure theory*, Springer, **1977**, pp. 1-27.

[5] a) V. Barone, M. Cossi, *J. Phys. Chem. A* **1998**, *102*, 1995-2001; b) M. Cossi, N. Rega, G. Scalmani, V. Barone, *J. Comput. Chem.* **2003**, *24*, 669-681.

[6] a) S. Grimme, J. Antony, S. Ehrlich, H. Krieg, *J. Chem. Phys.* **2010**, *132*; b) S. Grimme, *J. Comput. Chem.* **2004**, *25*, 1463-1473; c) S. Grimme, S. Ehrlich, L. Goerigk, *J. Comput. Chem.* **2011**, *32*, 1456-1465.

[7] M. C. D'Alterio, C. De Rosa, G. Talarico, *ACS Catalysis* **2020**, *10*, 2221-2225.

[8] P. Vermeeren, S. C. C. van der Lubbe, C. Fonseca Guerra, F. M. Bickelhaupt, T. A. Hamlin, *Nat. Prot.* **2020**, *15*, 649-667.

[9] E. D. Glendening, *J. Phys. Chem. A* **2005**, *109*, 11936-11940.

[10] E. D. Glendening, C. R. Landis, F. Weinhold, *J. Comput. Chem.* **2019**, *40*, 2234-2241.

[11] S. F. Boys, F. Bernardi, *Mol. Phys.* **1970**, *19*, 553-566.

[12] N. Nomura, R. Ishii, Y. Yamamoto, T. Kondo, *Chem. Eur. J.* **2007**, *13*, 4433-4451.
